# Electronic cigarettes: overview of chemical composition and exposure estimation

**DOI:** 10.1186/s12971-014-0023-6

**Published:** 2014-12-09

**Authors:** Jürgen Hahn, Yulia B Monakhova, Julia Hengen, Matthias Kohl-Himmelseher, Jörg Schüssler, Harald Hahn, Thomas Kuballa, Dirk W Lachenmeier

**Affiliations:** Chemisches und Veterinäruntersuchungsamt (CVUA) Sigmaringen, Fidelis-Graf-Straße 1, 72488 Sigmaringen, Germany; Chemisches und Veterinäruntersuchungsamt (CVUA) Karlsruhe, Weissenburger Strasse 3, 76187 Karlsruhe, Germany; Institute of Chemistry, Saratov State University, Astrakhanskaya Street 83, 410012 Saratov, Russia; Bruker Biospin GmbH, Silberstreifen, 76287 Rheinstetten, Germany

**Keywords:** Electronic cigarettes, Electronic nicotine delivery systems, Nicotine, Risk assessment

## Abstract

**Background:**

Electronic cigarettes (e-cigarettes) are advertised to tobacco users as a tool to decrease cigarette consumption and to reduce toxic exposure associated with conventional tobacco smoking. Little is known about the compounds contained in such products, their exposure and long-term health effects.

**Methods:**

NMR spectroscopy was used to ascertain the content of several constituents of e-cigarette liquids including nicotine, solvents and some bioactive flavour compounds. Risk assessment was based on probabilistic exposure estimation and comparison with toxicological thresholds using the margin of exposure (MOE) approach.

**Results:**

In 54 samples of e-cigarette liquids, the average nicotine content was 11 mg/ml. Only 18 from 23 samples were confirmed as nicotine-free samples and in one e-cigarette liquid nicotine was not detected while being declared on the labelling. Major compounds of e-cigarette liquids include glycerol (average 37 g/100 g), propylene glycol (average 57 g/100 g) and ethylene glycol (average 10 g/100 g). Furthermore, 1,3-propanediol, thujone and ethyl vanillin were detected in some samples. The average exposure for daily users was estimated as 0.38 mg/kg bw/day for nicotine, 8.9 mg/kg bw/day for glycerol, 14.5 mg/kg bw/day for 1,2-propanediol, 2.1 mg/kg bw/day for ethylene glycol, and below 0.2 mg/kg bw/day for the other compounds. The MOE was below 0.1 for nicotine, but all other compounds did not reach MOE values below 100 except ethylene glycol and 1,2-propanediol.

**Conclusions:**

NMR spectroscopy is a useful and rapid method to simultaneously detect several ingredients in e-cigarette liquids. From all compounds tested, only nicotine may reach exposures that fall into a high risk category with MOE <1. Therefore, e-cigarette liquid products should be subjected to regulatory control to ensure consistent nicotine delivery. Solvents with more favourable toxicological profiles should be used instead of ethylene glycol and 1,2-propanediol, which may fall into a risk category with MOE < 100.

## Background

An electronic cigarette (e-cigarette) is a part of an emerging class of electronic nicotine-delivery systems. These devices aerosolize nicotine (if contained) and produce a vapour that emulates that of tobacco cigarettes but purportedly has fewer traditional toxins than secondhand smoke [1]. The awareness about these products and availability of e-cigarettes on the Internet, including in Web searches, virtual user communities and online shops has increased dramatically in recent years [1,2], however, only few studies have been dedicated so far to the qualitative composition and toxicological characterization of these products [3,4].

The benefits and risks of electronic cigarette use are a subject of discussion among health organizations and researchers [5]. First, only limited studies have evaluated the effects of electronic cigarettes on human health. While e-cigarettes may have negligible influence on blood count indices, carbon monoxide exposure and heart rate [5-8], it was found that e-cigarettes may contain additional impurities in the liquids or vapour (e.g., polyaromatic hydrocarbons, aldehydes and acrolein), although at much lower concentrations than the ones found in normal cigarettes [9,10]. Furthermore, recent research suggests that these products may contain unexpected toxins and/or may provide unreliable nicotine delivery [1]. Finally, few and controversial empirical studies exist to determine whether e-cigarettes have potential as smoking cessation products [5,9,11].

In general, electronic cigarettes often contain ingredients such as propylene glycol, glycerol, ethylene glycol and polyethylene glycol mixed with concentrated flavours, and optionally, a variable percentage of nicotine [10,12-14]. Besides these major compounds, a number of other organic substances can be found in liquid formulated products and/or the vapour phase produced by an e-cigarette unit. These include tobacco specific nitrosoamines (TSNA) such as N-nitrosonicotine (NNN), N-nitrosoanabasine (NAB), N-nitrosoanabatine (NAT) and 4-(methylnitrosamino)-1-(3-pyridyl)-1-butanone (NNK) [10,12,14] or tobacco-specific impurities such as cotinine, anabasine and myosmine [12,13]. It was recently shown that e-cigarette liquids can be adulterated with pharmacologically active ingredients or their analogues such as rimonabant and amino-tadalafil [15].

Given the large increase in awareness and use of e-cigarettes and the unknown impact of their use on cigarette smoking behaviours and long-term health, development of analytical methodologies for these products is required. Previous research involved typically gas or liquid chromatography, combined with mass spectrometric detection for the analysis of such products, however, only a limited number of samples have been investigated in each particular study [13,14,16-20]. Considering high throughput including minimal sample preparation, fast spectra acquisition and processing, we hypothesized that direct nuclear magnetic resonance (NMR) spectroscopy might be applicable for a high throughput screening of e-cigarette liquids.

Some studies have found very inconsistent nicotine contents in some products regarding the labelling, i.e., e-cigarette liquids advertised as containing no nicotine may contain the substance and, in contrast, some products did not contain nicotine despite its presence being labelled [13,15]. Therefore, our study was principally aimed to develop a reliable and simple analytical tool based on NMR spectroscopy to control the absence/presence of nicotine in e-cigarette liquids. However, as the scope of quantitative NMR lies beyond quantification of a single analyte in a given matrix [21,22], we expanded our methodology also for the determination of other major compounds in e-cigarette liquids (solvents and flavour compounds). The procedure was then applied to analyse a large sample collection (n = 54) of e-cigarette liquids that have been bought over the Internet. Finally, the exposure of the consumer is estimated based on the analytical results and a toxicological assessment is provided.

## Methods

### Sampling and chemical analysis

A total of 54 e-cigarette liquid samples was analysed. This includes e-cigarette liquids (n = 20) submitted to the CVUA Karlsruhe for official medicines and tobacco control purposes in Baden-Württemberg, Germany. Furthermore, an internet-based market research was conducted to identify products offered to German consumers. To do this, we searched for the German terms “E-Zigaretten Liquids kaufen”, “E-Liquids Kaufen”, “E-Liquids Flash”, “E-Liquids Hanf”, “E-Liquids Marihuana” using the web platforms google.de, amazon.de and ebay.de. We also included search terms such as “E-Liquids Cialis”, “E-Liquids Vitamin“, because the German Federal Institute for Risk Assessment (BfR) recently found pharmacological active substances such as tadalafil, caffeine or vitamins in these products [23]. In total, 13 on-line shops were selected, that are selling such liquids. Most of the shops (n = 8) were located in Germany, one was located in Spain, three were located in the United Kingdom and one was located in Romania. Based on the invoices, it seems that several shops, though being located in countries outside Germany, had local partners, who handled the shipping and returning of the good, if necessary. The liquids were sent to us in envelopes or parcels. Most of the products were labelled as “Liquids for E-Cigarettes” or simply “E-Liquid”, but in some cases the products did not contain any labelling and in one case the products were labelled as “Air freshener”.

From the identified on-line shops, we selected samples using a risk-oriented approach. For example, we preferentially obtained samples where the presence of bioactive flavour compounds could be assumed. Additionally, several samples suspected of containing illegal or unusual substances were bought, such as products labelled as “Marihuana-Flavour”, “Mary Jane Flavour”, “Vitamin-Mix” or “Multi Vitamin”. Furthermore, several samples with tobacco and beverage flavour (such as cola, wine, energy drink or absinthe flavour) were included. We tried to obtain all varieties of e-cigarette liquids regarding declared nicotine content. We obtained several samples, claimed as nicotine-free and samples with medium or high nicotine content (the labelled nicotine content varied between 6 and 54 mg/ml).

### NMR analysis of electronic cigarette liquids

The chemical analysis was conducted using NMR spectroscopy based on a procedure previously developed for analysis of alcoholic beverages [24].

All solvents and reagents used were in pro analysis quality: nicotine, menthol, safrole (Sigma Aldrich, Steinheim, Germany), propylene glycol, 1,3-butanediol, 1,3-propanediol, ethylene glycol, glycerol, ethyl vanillin, camphor, α-thujone, coumarin, diethyleneglycol (Fluka, Buchs, Switzerland).

To obtain full quantitative information about e-cigarette liquids composition, we decided to apply two separate sample preparations for each sample. To measure water-soluble compounds (including nicotine), 60 μL of a sample was mixed with 480 μL of distilled water and 60 μL of NMR buffer (pH 7.4; 1.5 M KH_2_PO_4_ in D_2_O, 0.1 % 3-(trimethylsilyl)-propionate acid-d_4_ (TSP), 3 mM NaN_3_). Next, to get an overview of lipophilic substances, the following sample preparation was used: 100 μL of a sample was mixed with 800 μL CDCl_3_ containing 0.1% tetramethylsilane (TMS). After filtration (when necessary), 600 μL of the solution was poured into an NMR tube for direct measurement in both cases.

Stock standard solutions were prepared at a final concentration of about 10,000 mg/L in distilled water (nicotine bitartrate, propylene glycol, 1,3-butanediol, 1,3-propanediol, ethylene glycol, diethylene glycol and glycerol) or in deuterated chloroform (menthol, ethyl vanillin, coumarin, camphor, safrole and α-thujone). Calibration solutions were prepared by diluting the standard solutions in water or in deuterated chloroform and were measured as the authentic samples. The calibration curves were evaluated by integrating specific resonances of the selected compounds against TSP (in water) or TMS (in CDCl_3_) as an intensity reference.

All NMR measurements were performed on a Bruker Avance 400 Ultrashield spectrometer (Bruker Biospin, Rheinstetten, Germany) equipped with a 5-mm SEI probe with Z-gradient coils, using a Bruker Automatic Sample Changer (B-ACS 120). All spectra were acquired at 300.0 K.

NMR spectra of the aqueous solutions were acquired using Bruker standard water suppression 1D noesygppr1d pulse sequence with 64 scans (NS) and 4 prior dummy scans (DS). The sweep width (SW) was 19.9914 ppm and the time domain of the FID was 65536 (65 k). For the spectra acquisition of chloroform-dissolved samples, the Bruker experiment zg30 was used. After application of 2 dummy scans (DS), 8 free induction decays (FIDs) (NS = 128) were collected into a time domain of 131072 (131 k) complex data points using a 20.5503 ppm spectral width (SW) and a receiver gain (RG) of 101.

The data were acquired automatically under the control of ICON-NMR (Bruker Biospin, Rheinstetten, Germany), requiring about 40 min per sample for two measurements. All NMR spectra were phased and baseline-corrected.

The spectra of samples were compared to the spectra of the standards. Separated peaks corresponding to each substance were identified and integrated against TSP/TMS as an intensity reference using TopSpin v. 3.1 (Bruker Biospin, Rheinstetten, Germany). NMR ranges used for identification/integration for all compounds are listed in Table 1. Furthermore, all samples were screened for a range of small molecular weight molecules such as formaldehyde, acetaldehyde and acrolein.

**Table 1:** **Selected resonances, limits of detection (LOD) and limits of quantification (LOQ) for compounds in e-cigarette liquids**

	**Substance**	**Solvent**	**NMR range used for integration [ppm]**	**LOD [mg/L]**	**LOQ [mg/L]**
1	Nicotine	Water	8.68-8.60 (multiplet)	1.6	5.5
2	Propylene glycol (1,2-propanediol)		3.47-3.42 (multiplet)	2.1	6.9
3	1,3-Butanediol		1.75-1.70 (multiplet)	2.3	7.6
4	1,3-Propanediol		1.85-1.75 (multiplet)	0.96	3.2
5	Ethylene glycol		3.69-3.67 (singlet)	0.17	0.56
6	Diethylene glycol		3.78-3.73 (multiplet)	0.51	1.7
7	Glycerol		3.82-3.75 (multiplet)	2.6	8.7
8	Menthol	Chloroform	2.00-1.92 (multiplet)	12	40
9	Ethyl vanillin		9.81-9.83 (singlet)	1.0	3.4
10	Coumarin		7.80-7.65 (multiplet)	3.2	10
11	Camphor		2.40-2.30 (multiplet)	13	44
12	Safrole		6.80-6.60 (multiplet)	2.6	8.6
13	Thujone (sum of α- and β-diastereomers)		2.12-2.09 (singlet)	3.4	11

We conducted a detailed validation for the determination of nicotine in e-cigarette liquids. For this substance the limits of detection (LOD) and quantification (LOQ) were calculated from the residual standard deviation of the regression line [25]. To assess reproducibility, standard solutions as well as an e-cigarette liquid sample were analyzed several times daily (*n* = 5). The recovery rate was ascertained by adding nicotine standard solution at four different concentrations to a real sample.

For other substances, the LOD and LOQ values were determined as signals for which the signal-to-noise ratios (SNR) are 3 and 10, correspondingly. SNR were calculated using the Bruker *sino* routine implemented in the Topspin 3.1 software package (Bruker Biospin, Rheinstetten, Germany). The limits of the noise and signal regions were located near to each other and were set manually for each spectrum.

The linearity of the calibration curves was evaluated in the range covering the concentrations found in the investigated products.

### Risk assessment

The methodology for comparative quantitative risk assessment was based on a previous study conducted for compounds in alcoholic beverages [26] with the exception that probabilistic exposure estimation was conducted [27-29].

The toxicological thresholds, preferably benchmark doses (BMD) or if unavailable no observed effect levels (NOEL), no observed adverse effect levels (NOAEL) or lowest observed adverse effect levels (LOAEL), for the selected substances were typically identified in monographs of national and international risk assessment bodies such as EFSA, OECD SIDS, JECFA, and ATSDR [30-35], and if unavailable – as in the case of thujone - taken from an own study [36].

The MOE approach was used for risk assessment [37,38]. The MOE is defined as the ratio between the lower one-sided confidence limit of the BMD (BMDL) or NOEL/NOAEL/LOAEL and estimated human intake of the same compound.

The exposure was estimated for daily users of e-cigarettes based on the contents found in our chemical analysis. Similar to the approach of Medeiros Vinci et al. [39] for probabilistic human exposure assessment of food contaminants, best fit distributions were applied to the substance contents and the resulting risk functions were entered into the probabilistic analysis. Further assumptions were literature data about typical e-cigarette liquid use per day [40] and literature data about vaporization percentage [19]. The bodyweight was assessed as normal distribution with average of 73.9 kg and standard deviation of 12 kg for males and females [41]. All risk functions were truncated at zero because negative values are factually impossible. Monte Carlo simulations were performed with 10,000 iterations using Latin Hypercube sampling and Mersenne Twister random number generator. Calculations were performed using the software package @Risk for Excel Version 5.5.0 (Palisade Corporation, Ithaca, NY, USA).

## Results

### NMR method development and validation results

**Figure 1:**
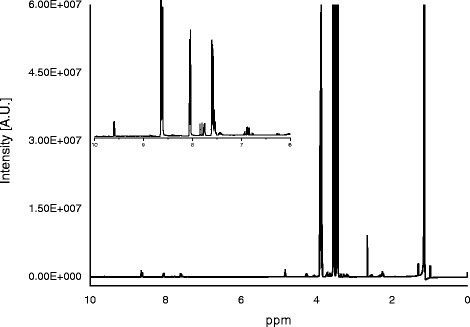
^**1**^ **H NMR spectrum of the aqueous sample of a typical e-cigarette liquid sample.** The insert shows ^1^H NMR spectra in the 10–6 ppm region.

Figure 1 shows the complete ^1^H NMR spectrum of a sample measured in aqueous solution of an e-cigarette liquid sample as well as a magnification of the δ 10–6.0 ppm region, where the resonances of aromatic protons (for instance, from nicotine and ethyl vanillin) can be observed. The mid-field region of NMR spectra contains information about major solvents such as glycerol, propylene glycol and ethylene glycol. NMR spectra of chloroform-dissolved samples have the same principal signals, however, some additional non-polar compounds (e.g. thujone, camphor) can be seen.

**Figure 2:**
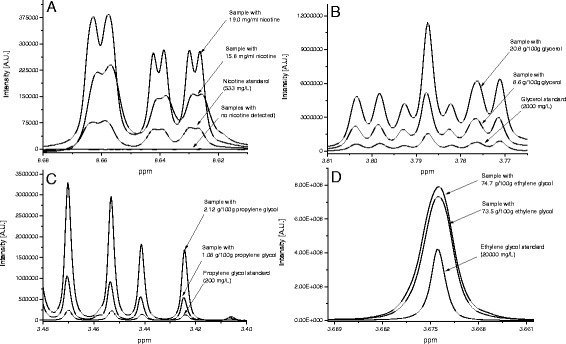
**NMR resonances of nicotine (A), glycerol (B), propylene glycol (C), ethylene glycol (D) in standard solutions and e-cigarette liquid samples**

The NMR ranges (i.e. peaks not overlapped or interfered by matrix) selected for quantification are given in Table 1. For example, nicotine, the principal compound of interest in our research, has signals at δ 8.65 ppm (m), δ 8.07 ppm (m), δ 7.60 ppm (m), δ 4.39 ppm (m), δ 4.32 ppm (s), δ 3.80 ppm (m), δ 3.30 ppm (m), δ 2.73 ppm (s), δ 2.68 ppm (m) and δ 2.38 ppm (m). However, the resonances in the aliphatic and mid-field ranges were found unsuitable for quantification because they showed strong overlap with other matrix compounds (Figure 1). Considering the signals in the aromatic region, we decided to use the multiplet at δ 8.65 ppm for quantification because this leads to the best sensitivity and this signal was not interfered in any case. As an example, Figure 2A shows the signal of nicotine in standard solutions and in four e-cigarette liquid samples (from these, two products contained 19.0 mg/ml and 15.8 mg/ml nicotine and the other two contained not detectable nicotine concentrations). For the other substances, signals not interfered by matrix were also identified (Table 1) and as example, spectra for glycerol, propylene glycol and ethylene glycol in standard solutions as well as in samples are presented in Figure 2B-D.

Table 2 summarizes the NMR method validation results for nicotine. The ^1^H NMR assay was linear in a working concentration range of 5–10,000 mg/L (R = 0.9992), which means 0.050-100 mg/ml e-cigarette liquid considering sample preparation. This range basically covers the levels of nicotine in e-cigarette products and no further dilution/extraction of the matrix is required. The LOD and LOQ were 1.6 and 5.5 mg/L, correspondingly, which is equal to 0.016 and 0.055 mg/ml e-cigarette liquid. We propose to consider our LOD value as a cut-off level, at which we can distinguish nicotine-free and nicotine-containing products.

**Table 2:** **Results of method validation for nicotine**

**Parameter**		**Result**
Linear range		5-10,000 mg/L (0.050- 100 mg/ml sample)
LOD^a^		1.6 mg/L (0.0157 mg/ml sample)
LOQ^a^		5.5 mg/L (0.0546 mg/ml sample)
Recovery		99% (at 1000 mg/L)
		104% (at 1500 mg/L)
		115% (at 1750 mg/L)
		108% (at 2000 mg/L)
		107% (average)
Variation coefficient (n = 5)	Standard solution	1.2 %
	E-cigarette liquid sample	2.1 %

^a^Limit of detection (LOD) and quantitation (LOQ) were determined by establishing a separate calibration curve near LOD (5.0-25 mg/L). The limits were calculated from the residual standard deviation of the regression line [25].

Furthermore, Table 1 contains LOD and LOQ values for all other compounds (except nicotine). The LOD values varied in the 0.17 - 13 mg/L range with the lowest values for glycols (for ethylene glycol 0.17 mg/L and for diethylene glycol 0.51 mg/L) and highest for camphor (13 mg/L) and menthol (12 mg/L) (Table 1). The LOQ values were in the range between 1.7 mg/L (ethylene glycol) and 40 mg/L (menthol). The high correlation coefficients (R > 0.99) obtained for the calibration graphs indicate a good linear response within the concentration range studied. From fifty four samples investigated only in five we were not able to directly quantify glycerol, ethylene glycol or propylene glycol due to spectral overlap.

The method was further validated by repeated sample preparation of a standard solution (1000 mg/L) and an e-cigarette liquid sample (nicotine content 19.0 mg/ml). Recoveries between 99% and 115% (average 107%) were obtained when the standard addition method is applied for an e-cigarette liquid sample. The variation coefficients were found to be 1.2% for the standard solution and 2.1% for the product. In general, the results of the method validation show that the method is sufficiently precise and reproducible and is adequate for the purpose of regulatory control of e-cigarette liquids (Table 1).

Moreover, reference HPLC analysis was performed for a subgroup of 15 samples (Table 3). Statistical analysis between results from the two methods (HPLC and NMR) revealed that the linear correlation is significant (ANOVA *p* < 0.0001, R = 0.98). No systematic or proportional differences between both methodologies were found, as the standard deviations of both the y-axis and the slope were encompassing 0 or 1. The results confirmed the accuracy of our NMR method and its comparability with HPLC.

**Table 3:** **Comparison of nicotine quantification results between NMR and HPLC methods**

**Sample number**	**Nicotine content [mg/ml]**	
	**NMR**	**HPLC**
1	19.0	17.9
2	15.8	17.2
3	17.7	16.3
4	21.0	17.6
5	n.d.	n.d.
6	n.d.	n.d.
7	n.d.	n.d.
8	n.d.	n.d.
9	n.d.	n.d.
10	13.7	13.9
11	23.5	22.5
12	14.3	17.0
13	24.3	22.9
14	14.1	17.5
15	18.7	17.5

Finally, we have also compared our NMR results with the reference GC-MS values for a set of 10 samples. It was found that the data of both methods are in good agreement with each other: the relative difference varied between 1.7% and 13% (average 8.1%) for 1,2-propanediol, between 2.8% and 7.3% (average 5.3%) for ethylene glycol and between 0.45% and 12% (average 7.5%) for glycerol.

### Composition of e-cigarette liquids

The NMR methodology was used for analysis of 54 authentic liquids for use in e-cigarettes. The results of our investigations are summarized in Table 4. From the analysed 54 samples, 34 (63%) contained nicotine above the detection limit. The average and median concentrations of nicotine in all investigated samples were 11 and 6.8 mg/ml, correspondingly (Table 4). In general, the values were in agreement with labelling. However, from 23 samples that were declared as nicotine-free, only 18 were confirmed to contain undetectable nicotine concentrations by NMR. Nicotine was definitely detected in 5 allegedly “nicotine-free” samples in a concentration range from 0.11 mg/ml to 6.9 mg/ml. In contrast, one e-cigarette liquid sample did not contain nicotine, while its presence being declared on the labelling (12 mg/ml).

**Table 4:** **Overview about constituents in electronic cigarettes with descriptive statistics and best fit distributions**

**Agent**	**Positive samples**	**Mean**	**Median**	**Standard deviation**	**Best fitting risk function for concentration of agent in the beverage** ^**a**^
Nicotine (mg/ml)	65%	11	6.8	13	*RiskNormal*(11.023;13.134;*RiskTruncate*(0;))
Glycerol (g/100 g)	94%	37	35	23	*RiskWeibull(1.8104;44.812;RiskShift(−2.8327);RiskTruncate(0;))*
1,2-Propanediol (g/100 g)	94%	57	64	30	*RiskTriang(−18.939;91.8;100.45;RiskTruncate(0;))*
Ethylene glycol (g/100 g)	91%	10	5	18	*RiskLoglogistic(−0.40204;5.15;1.8215;RiskTruncate(0;))*
1,3-Propanediol (g/100 g)	13%	0.6	0	1.7	*RiskResample(2;[all measurements])*
Thujone (mg/L)	4%	6.7	0	34	*RiskResample(2;[all measurements])*
Ethyl vanillin (mg/L)	26%	30	0	68	*RiskResample(2;[all measurements])*

^a^The best fit distributions were selected based on Kolmogorov-Smirnov statistics. For 1,3-propanediol, thujone and ethyl vanillin, distribution fitting was not possible due to the low incidence. Random resampling is used for these agents from a data table with all measurements where all samples with not detectable concentrations were treated as zero.

Glycerol and propylene glycol were detected in all samples at concentrations ranging from 0.3 to 95 g/100 g (average 37 g/100 g) for glycerol and from 0.4 to 98 g/100 g (average 57 g/100 g) for propylene glycol. Generally, lower levels of another solvent ethylene glycol (average 10 g/100 g) were detected. 1,3-Propanediol was detected only in 7 samples in the concentration range of 3.3-10 g/100 g. 1,3-Butanediol, diethylene glycol, formaldehyde, acrolein, coumarin, camphor, safrole and menthol were negative in all samples.

The presence of the major compounds glycerol and propylene glycol corresponded to the labelling in the majority of cases, except 3 products contained no labelling information at all. Glycerol was not labelled on 5 products despite being present. Propylene glycol was not labelled in 2 products despite being present. In one case, “vegetal glycol” was labelled without specifying the exact chemical compound. Ethylene glycol was not labelled on any of the samples, which did contain the compound.

As for flavour compounds, which can be monitored in NMR spectra of chloroform-dissolved samples, we detected thujone (the sum of α-/β-diastereomers) in two samples (183 and 178 mg/L) and ethyl vanillin (concentration range 7.7-335 mg/L) in thirteen samples. We observed coumarin, camphor, safrole and menthol in none of the samples (the presence of menthol was not labelled on any of the samples, however). Interestingly, among other volatile aldehydic compounds (acetaldehyde, formaldehyde and acrolein) we observed only acetaldehyde in one sample at a concentration of about 38 mg/L. Only 4 products contained a detailed ingredients list with specific flavour compounds pointed out. Most of the products labelled only “flavour” or “natural and artificial flavours” without pointing out specific substances, or completely lacked any labelling.

### Risk assessment for daily users of e-cigarettes

Nicotine, glycerol, 1,2-propanediol, ethylene glycol, 1,3-propanediol, thujone and ethyl vanillin were selected for exposure assessment. An overview of the concentrations of these compounds in e-cigarette liquids as well as the best fitting risk functions that were selected as input for probabilistic modelling are provided in Table 4. The toxicological thresholds are shown in Table 5 [30-36,42-48]. To provide a conservative assessment, the most sensitive toxicological endpoint was chosen, when several endpoints were available. Only for nicotine, human data were available as basis for the assessments. For the rest of the compounds, the assessments have to be based on animal data. The thresholds of the compounds vary over a very wide range, from 0.008 mg/kg bodyweight(bw)/day for nicotine to 10,000 mg/kg bw/day for glycerol.

**Table 5:** **Toxicological thresholds selected for calculating the margin of exposure**

**Agent**	**Toxicological Endpoint** ^**a**^	**Value [mg/kg bw/day]**	**Type of endpoint** ^**b**^	**Reference**
Nicotine	Heart rate acceleration in humans	0.008	LOAEL^c^	EFSA [30] based on Lindgren et al. [42]
		0.0008	ADI	
Glycerol	2-year study in rats, no effects observed	10,000	NOAEL	OECD SIDS [31] based on Hine et al. [43]
1,2-Propanediol	2-year studies in rats and dogs, no effects observed in rats, increased erythrocyte destruction in dogs	2,500	NOAEL	JECFA [32] based on Gaunt et al. [44] and Weil et al. [45]
		25	ADI	
Ethylene glycol	Developmental toxicity data in mice (total malformations and a skeletal variation)	76	BMDL_10_	ATSDR [33] based on Neeper-Bradley et al. [46] and Tyl et al. [47]
		0.8	MRL	
1,3-Propanediol	Developmental toxicity study in rats	1,000	NOAEL	EFSA Panel on Contaminants in the Food Chain [34] based on unpublished data
Thujone	Clonic seizures in rats	11	BMDL_10_	Lachenmeier and Uebelacker [36] based on NTP [48]
		0.11	ADI	
Ethyl vanillin	13-week study in rats, no effects observed	500	NOEL	JECFA [35] based on unpublished data
		3	ADI	

^a^Human data was preferred over animal data, if available. The most sensitive endpoint was chosen if dose–response data for several organ sites were available.

^b^BMDL_10_: lower one-sided confidence limit of the benchmark dose (BMD) for a 10% incidence of health effect. The No Effect Level (NOEL) or No Observed Adverse Effect Level (NOAEL) are used in cases when no usable BMD-modelling for oral exposure was identified in the literature. ADI: acceptable daily intake. MRL: minimal risk level.

^c^The lowest observed adverse effect level (LOAEL) is considered by EFSA [30] as close to the NOAEL. The values were derived from a study in humans who were injected nicotine intravenously assuming an oral bioavailability of 44%.

Table 6 presents the probabilistic exposure estimates. In all cases, the highest exposure was detected for 1,2-propanediol (average 14.5 mg/kg bw/day), while the lowest was found for thujone (average 1.6E-04 mg/kg bw/day).

**Table 6:** **Estimated exposure (mg/kg bw/day) of electronic cigarette users using Monte Carlo analysis (10,000 iterations)** ^**a**^

**Agent**	**Mean**	**SD**	**Median**	**P5**	**P95**
Nicotine	0.38	0.39	0.25	0.02	1.15
Glycerol	9.0	8.9	6.2	0.6	27.2
1,2-Propanediol	14.5	12.4	11.0	1.3	39.3
Ethylene glycol	2.1	6.7	0.9	0.1	7.1
1,3-Propanediol	0.14	0.51	0	0	1.1
Thujone	1.6E-4	9.7E-4	0	0	Max 0.01
Ethyl vanillin	7.2E-4	2.0E-3	0	0	Max. 0.03

^a^Calculated for all agents using the following formula with the risk functions defined in Table 4:

Exposure = Risk function of concentration * Risk function of e-cigarette liquid amount per day * Risk function of vaporization percentage / Risk function of bodyweight (kg).

The risk function of e-cigarette liquid amount per day was *RiskNormalAlt(25%;3;75%;5;RiskTruncate(0;))* based on values from Farsalinos et al. [40]

The risk function of vaporization percentage was RiskUniform(6;81) based on values from Goniewicz et al. [19].

The risk function of bodyweight was *RiskNormal(73.9;12)* according to average and standard deviation from EFSA Scientific Committee [41].

The analysis also includes a sensitivity analysis, which allows a ranking of the input distributions which impact the exposure. In all cases, the concentration of the compound in the liquid had the highest influence, followed by vaporization percentage, e-cigarette liquid amount per day and a minor influence of bodyweight (regression coefficients for concentration ranging between 0.58 and 0.87, for vaporization percentage between 0.08 and 0.50, for e-cigarette liquid amount per day between 0.04 and 0.43 and for bodyweight between −0.02 and −0.17).

**Figure 3:**
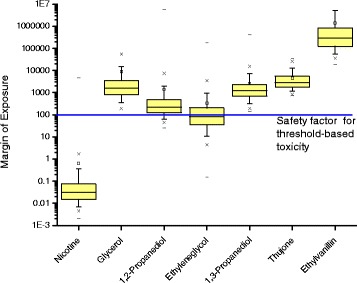
**Margin of Exposure (MOE) for compounds occurring in electronic cigarettes based on probabilistic exposure estimation (simulation with 10,000 iterations).** (The box is determined by the 25^th^ and 75^th^ percentiles. The whiskers are determined by the 5^th^ and 95^th^ percentiles. 1^st^ and 99^th^ percentiles are marked by x, while minimum and maximum are marked with dash. Values above 1E7 are not shown).

Finally, the margins of exposure (MOE) for all compounds are compared in Figure 3. Nicotine is the only compound, for which the complete distribution is below a MOE of 10, and on average below 0.1. From all other compounds, only ethylene glycol may reach MOEs below 100 in about 50% of cases and 1,2-propanediol in worst cases (above the 75th percentile).

## Discussion

### Chemical analysis of e-cigarette liquids

Regarding nicotine quantification in e-cigarette liquids, several analytical methodologies are available, including HPLC-UV [12,13,17,49], headspace GC-MS [12], GC-FID [50], and GC-TID (Thermionic specific detector) [19]. As expected, our NMR method with a limit of detection (LOD) of 1.6 mg/L is not as sensitive as these methods (for example, the LOD of HPLC was about 0.1 mg/L [13]), however it is sufficient to control the nicotine content in nicotine-containing e-cigarette liquids (Table 1).

For the identification of the major ingredients (glycols and glycerol) and their relative concentrations, gas chromatography with flame ionization detector (GC-FID) or with mass spectrometry (MS) is usually used [50,51]. These methods provide adequate sensitivity, but have the disadvantage of being very laborious and time-consuming. Moreover, some authors noted a significant matrix effect, which results in peak suppression of analytes [10,14]. This matrix effect effectively limits the amount of propylene glycol and glycerol in the measurement solution and necessitates dilution of the samples with subsequent rise in LOD. For NMR, we found that a dilution factor of 10 is sufficient to obtain good phase- and baseline corrected spectra, which still provides sufficient sensitivity for all compounds in e-cigarette liquids. Thus, our results have shown that NMR is a good alternative for the control of nicotine and a number of other compounds in e-cigarette liquids and it avoids the use of two separate analytical techniques (HPLC for nicotine and GC for volatiles).

^1^H-NMR spectroscopy can therefore provide quantitative information necessary to judge about the composition of e-cigarette products in a short analysis period (about 20 minutes including sample preparation). First and foremost, we can provide very fast binary categorization, if an e-cigarette liquid sample contains nicotine or not (LOQ is about 0.050 mg/ml e-cigarette liquid) and, therefore, control the labelling. This is not only required for market control of the products but also important for consumer health protection as an e-cigarette user could be exposed to the hazard of nicotine dependence by purchasing a product, which while advertised as containing no nicotine does contain it. Moreover, all major compounds could be identified and quantified using the same spectral data. In case of spectral overlap, multivariate deconvolution methods can be supplementarily used [52].

Similar to our findings, other authors have also observed that the nicotine content of some e-cigarette liquids is often inconsistent with the labelling [13,15].

Acetaldehyde, which we found in one sample, has been also recently detected in one e-cigarette liquid sample by Selected Ion Flow Tube Mass Spectrometry (SIFT-MS) [10]. However, we think that it is currently not possible to estimate acetaldehyde exposure by e-cigarette consumption based on these single observations.

In our study we detected none of the tobacco-specific impurities and tobacco specific nitrosamines by NMR (data not shown). However, as these compounds are usually found in trace concentrations, the sensitivity of NMR is not sufficient to control the typical levels of these compounds in e-cigarette liquids [10]. For the analysis of tobacco-specific impurities and tobacco specific nitrosamines, hyphenated methods such as HPLC-MS/MS, GC-MS, headspace GC-MS or GC-MS/MS methods have to be used [10,12,14,16,49]. Even applying these methods, concentrations were usually found to be below LOQ levels [10,12]. Tobacco-specific toxicants and trace nicotine impurities were judged as being below levels likely to cause harm [17,20].

For our risk assessment, we have therefore focused on the constituents with major occurrence in e-cigarette liquids (nicotine, glycerol, 1,2-propanediol, and ethylene glycol). 1,3-Propanediol, thujone and ethyl vanillin were included as compounds of minor occurrence, but which could be relevant for consumers that prefer single brands of products in light of a worst-case scenario.

### The risk of compounds in e-cigarette liquids

Exposure estimates may have considerable uncertainty especially in the case of non-normal distributions, as in our case of constituents in e-cigarette liquid. For this reason, we decided to apply a probabilistic method, which takes account of every possible value that each variable can take and weight each possible scenario by the probability of its occurrence [53]. To facilitate this, we use the Monte-Carlo approach, which has been previously applied in food science to model dietary exposure to chemicals in food [39,53,54], but this study is the first to apply it to estimate the exposure to chemicals in tobacco related products. The advantage of the approach is that rather than single values for each scenario it generates distributions of the MOE, which allow a direct visualization and comparison of all scenarios (Figure 3). In line of a worst case scenario, we assume that the vaporized percentage of the liquid may have a 100% bioavailability either orally or inhalatory. As toxicological thresholds for inhalation exposure were unavailable, we use thresholds for oral exposure, which may be seen as a limitation of the approach.

According to the typical interpretation of MOEs derived from animal experiments (i.e. for all our compounds except nicotine), MOE < 10 is judged to pose “high risk”, while MOE < 100 are judged as “risk”. MOEs above 100 are often judged as acceptable because the value of 100 corresponds to the default 100-fold uncertainty factor, which has been historically used in regulatory toxicology. The factor of 100 is based on scientific judgement and represents the product of two separate 10-fold factors that allow for interspecies differences and human variability [38,55]. When the toxicological endpoint is based on human data on not on animal experiments as for nicotine, MOEs above 10 would be judged acceptable and MOEs below 1 as “high risk”. Using this interpretation of MOE, our evaluation clearly shows that nicotine is by far the compound with the highest risk in e-cigarette liquids. The MOE values of nicotine are all below 10 and may reach down to below 0.1, which is the lowest level of all compounds under study (Figure 3). Nicotine exposure would also exceed the ADI as proposed by EFSA [30] for nicotine residues in food products. Both 1,2-propanediol and ethylene glycol may reach MOEs below 100 in some scenarios, which may be interpreted as falling into the “risk” category.

Daily users of conventional cigarettes may have acquired a very high tolerance to nicotine, so that our assessment may overestimate the risk of this user group. An overestimation of the risk of nicotine may also occur by the endpoint of heart rate acceleration, which was selected by EFSA in their risk assessment of nicotine in foods [30]. It may be questioned if heart rate acceleration is a suitable (adverse) endpoint for risk assessment. The acute risk of nicotine may have been generally overstated in the past [56]. On the other hand, the chronic risk of nicotine may not be adequately considered by the endpoint chosen by EFSA [30] as well. For example, some new *in vitro* evidence points to possible direct carcinogenic and genotoxic effects of nicotine [57-60]. Nevertheless, epidemiological evidence for such an effect of nicotine appears to be unavailable so far; and while being on the “high priority” list for evaluation by the WHO International Agency for Research on Cancer, nicotine has so far not been classified by the agency.

In consideration of the potential over- or underestimation of the effects of nicotine, we agree with EFSA [30] to apply the heart-rate acceleration as most sensitive human endpoint till better data become available. Our risk assessment provides evidence that non-tolerant users of e-cigarettes should be clearly warned against using liquids with higher nicotine contents. It should also be noted as limitation that the potential hazard of nicotine dependence, which may depend on the form and speed of nicotine delivery [61], currently cannot be considered due to lack of adequate dose–response data.

Average ethylene glycol exposure would also exceed the minimal risk level of ATSDR [33]. It should be also noted that ethylene glycol (in contrast to propylene glycol) is not included on the U.S. Food and Drug Administration list of compounds “generally recognized as safe” (GRAS) [62].

Nevertheless, we think that compared to nicotine, the risks of 1,2-propanediol and ethylene glycol appear to be minor. However, the difference is that nicotine is intentionally ingested while the consumers certainly believes that all other constituents of the liquids are without risk (especially considering the advertisement of e-cigarettes as risk-free alternatives to tobacco cigarettes). For this reason in a conservative assessment, prudent risk management could be to avoid or reduce these compounds in the liquids and switch the formulations to other solvents with more favourable toxicological profiles. The risk of the flavour compounds thujone and ethyl vanillin appears to be rather low (MOE >1000) and mitigation measures for toxicological reasons appear to be not required.

## Conclusions

E-cigarettes are a class of products that has emerged to the mass market during the last decade and the regulatory status of which has been unclear in a borderline area between regulations for medicinal products, tobacco products or general product safety rules.

One possibility would have been to regulate e-cigarettes as medicinal product. Our research clearly shows that the exposure of nicotine may reach amounts that facilitate a pharmacological action in the user of the product in the sense of the European medicines directive [63]. However, there was a political decision against this option. Such products will be regulated in the context of a revised tobacco products directive in the European Union, which was formally approved by the European Parliament on 26 February 2014. The maximum nicotine threshold will be 20 mg/ml [64].

We fully agree with the intention of the regulation to ensure uniform within-device dosing (i.e. the same product should always be consistent in nicotine delivery, which may be achieved by quality control of the liquids as well as by ensuring consistent nicotine delivery from the e-cigarette device) [65]. Our study has shown that deviations from the labelling in certain products would demand quality control procedures by official authorities similar to other consumer products such as alcohol and tobacco cigarettes. Liquids with high nicotine levels may pose risks especially for non-tolerant users (i.e. users that have not smoked before). Furthermore, the vaporization percentage of the device has an important influence on the exposure, so that the dosing in the device itself must be controlled for consistency.
